# Prevalence of multidrug-resistant and extended-spectrum β-lactamase producing *Escherichia coli* from local and broiler chickens at Cibinong market, West Java, Indonesia

**DOI:** 10.14202/vetworld.2024.179-184

**Published:** 2024-01-22

**Authors:** Syaiful Rizal, Intan Nurhapsari, Ima Fauziah, Masrukhin Masrukhin, Yoga Dwi Jatmiko

**Affiliations:** 1 Research Center for Applied Zoology, Research Organization for Life Science and Environment, National Research and Innovation Agency (BRIN), Indonesia, Soekarno Science and Techno Park, Jl. Raya Jakarta - Bogor KM. 46, Cibinong, Bogor 16911, West Java, Indonesia; 2 Department of Biology, Faculty of Mathematics and Natural Sciences, Universitas Brawijaya, Jl. Veteran, Malang 65145, East Java, Indonesia; 3 Research Center for Biosystematics and Evolution, Research Organization for Life Science and Environment, National Research and Innovation Agency (BRIN), Indonesia, Soekarno Science and Techno Park, Jl. Raya Jakarta - Bogor KM. 46, Cibinong, Bogor 16911, West Java, Indonesia

**Keywords:** antibiotic, chicken, extended-spectrum beta-lactamase, *Escherichia coli*, multidrug resistance

## Abstract

**Background and Aim:**

Antimicrobial resistance (AMR) is becoming a public health concern. Foodborne pathogens are infectious agents that can be transmitted from animals to humans through food and can become resistant due to misuse and overuse of antibiotics, especially in poultry. This study aimed to detect the prevalence of multidrug-resistant and extended-spectrum β-lactamase (ESBL)-producing *Escherichia coli* isolated from local and broiler chickens at the Cibinong market, West Java, Indonesia.

**Materials and Methods:**

A total of 60 cloacal swab samples from 30 local and broiler chickens sold at the Cibinong market in West Java were obtained by random sampling. From these samples, 39 *E. coli* isolates were obtained after being cultured on eosin methylene blue agar and molecularly identified using polymerase chain reaction (PCR). Six antibiotic disks were used for the antibiotic sensitivity test against *E. coli* isolates cultured on Mueller-Hinton agar. PCR was performed to detect ESBL genes (*bla*TEM, *bla*SHV, and *bla*CTX-M).

**Results:**

A total of 76.47% (39/51) cloacal swab samples were positive for *E. coli*. All *E. coli* isolates were sensitive to imipenem (100%), and 38 isolates were sensitive to cefoxitin (FOX) (97.4%). On average, the isolates were sensitive to amoxicillin-clavulanic acid (AMC) (69.2%) and ceftriaxone (CRO) (89.7%). *E. coli* isolates were occasionally resistant to enrofloxacin (25.64%), followed by gentamicin (20.51%), CRO (10.25%), AMC (7.69%), and FOX (2.56%). The prevalence of *E. coli* AMR was 10.25% (4/39). All four multidrug-resistant *E. coli* isolates (*bla*TEM and *bla*CTX-M) were confirmed to have the ESBL gene based on PCR.

**Conclusion:**

The prevalence of multidrug-resistant and ESBL-producing *E. coli* is still found, proving that there is still inappropriate use of antibiotics and a need for strict supervision of their use, especially around Cibinong market, West Java.

## Introduction

Antibiotics commonly used in human medicine and animal husbandry have increased antibiotic resistance, resulting in global health problems [1]. It is caused by misuse and overuse of antibiotics [2]. The World Health Organization (WHO) has identified antibiotic resistance as one of the top health risks of the 21^st^ century, with approximately 1.2 million people killed each year, especially in developing countries, including Indonesia [3, 4]. Poultry farms contribute to antibiotic resistance because inappropriate use of antibiotics is often found in chicken farms [5]. Antibiotics are usually used for treating and preventing bacterial infections and growth boosters, especially in poultry production [5]. Although antibiotic growth promoters have been banned by the Indonesian government, farmers continue to use them because of the low level of supervision by the authorities [6]. Antibiotic-resistant bacteria may indirectly reach humans along the food chain through the consumption of contaminated food or food-derived products and following direct contact with colonized/infected animals or biological substances, such as blood, urine, feces, saliva, and semen [7]. In addition, if antibiotic-resistant bacteria in the human body lead to infection and spread to other people, public health problems may occur. As a result, the treatment and healing process with certain antibiotics becomes ineffective [8].

Indonesia has a higher antimicrobial resistance (AMR) level than Australia and New Zealand [9, 10]. Furthermore, according to the global AMR surveillance system 2021 report by the WHO [11], there was an increase in AMR bloodstream infections (BSIs) of *Escherichia coli* and *Salmonella* spp. with an increased rate of 15% compared to 2017 in 99 countries, territories, and regions. Several types of pathogenic bacteria cause BSIs, one of which is *E. coli* [11]. The presence of *E. coli* is a sign of hygiene and sanitation. Cibinong Market, located in Bogor Regency, West Java, is a traditional wet market that sells various goods such as vegetables, fruits, fish, meat, and live birds. Livestock poultry, such as broiler chickens and local chickens imported from different areas, are sold there. As a result, assessing zoonotic *E. coli* AMR in the Cibinong market will help us understand the state of antibiotic contamination in the animal environment and serve as a reference for developing livestock husbandry rules and conserving the natural environment in Bogor Regency.

This study aimed to determine the presence of antibiotic-resistant and extended-spectrum β-lactamase (ESBL) *E. coli* in local and broiler chicken sold at the Cibinong market to ensure public health.

### Materials and Methods

#### Ethical approval

Ethical approval was not necessary for this study. Cloacal swab samples were collected shortly after the seller sold the chickens. However, samples were collected aseptically in accordance with established collection methods.

### Study period and location

This study was conducted from January 2022 to March 2023 at Indonesian Culture Collection (InaCC), National Research and Innovation Agency (BRIN), Bogor Regency, West Java, Indonesia.

### Sample collection, and bacterial isolation, and identification

Using a collection swab (Citoswab™, Jiangsu, China), samples of cloacal swabs were randomly taken from 30 local chickens and 30 broilers sold at the Cibinong market. After the samples were aseptically collected, they were immediately sent to the InaCC for bacteriological analysis. Citoswab™ was cultured on eosin methylene blue agar (EMBA, Himedia™, Mumbai, India) and incubated at 37°C for 24–48 h to identify the *E. coli* strains. After the incubation period, a green metallic sheen with black center colonies was assumed to be *E. coli*. Therefore, molecular confirmation using polymerase chain reaction (PCR) by *usp*A gene detection is necessary for identification [12].

### Antimicrobial susceptibility test

Confirmed *E. coli* isolates were subsequently cultured on Mueller-Hinton agar (MHA, Oxoid™, Basingstoke, England) subsequently. A bacterial suspension with turbidity equal to the 0.5 McFarland standard was readied and evenly spread on (MHA, Oxoid™). Antibiotic disks were placed on the agar surface. Subsequently, it was incubated at 37°C for 16–18 h. Amoxicillin-clavulanic acid (AMC, 30 μg), ceftriaxone (CRO, 30 μg), cefoxitin (FOX, 30 μg), imipenem (IPM, 10 μg), enrofloxacin (ENR, 5 μg), and gentamicin (CN, 10 μg) were used as antibiotic disks. The antimicrobial sensitivity test was performed according to the recommendations of the Clinical Laboratory Standards Institute [13]. We examined the inhibitory zone and measured its diameter using a caliper. *E. coli* ATCC 25922, considered as the positive control, and *Staphylococcus aureus* ATCC 25923, designated as the negative control, were employed to evaluate the susceptibility classification (sensitive, intermediate, or resistant).

### ESBL gene detection

The multidrug-resistant *E. coli* isolates were then tested for resistance genes *bla*TEM, *bla*CTX-M, and *bla*SHV using a polymerase chain reaction [12, 14]. Table-1 shows the sequences of the oligonucleotide primers and the size of the PCR products. PCR was performed in a total volume of 30 μL consisting of 1.5 μL for each forward and reverse primer, 2 μL DNA template, 15 μL MyTaq HS red mix (Bioline GmbH, Germany), and 10 μL nuclease-free water (Thermo Fisher Scientific™, USA).

**Table-1:** Primers used for the detection of *Escherichia coli* and ESBL genes.

Gene	Primer sequences	Size (bp)	Reference
*usp*A-F	5’- CCGATACGCCTGCCAATCAGT-3’	884	[12]
*usp*A-R	5’- ACGCAGACCGTAGGCCAGAT-3’		
*bla*TEM-F	5’- GCGGAACCCCTATTTG	964	[15]
*bla*TEM-R	3’- ACCAATGCTTAATCAGTGAG-5’		
*bla*SHV-F	5’- TTATCTCCCTGTTAGCCACC-3’	795	[14]
*bla*SHV-R	5’- GATTTGCTGATTTCGCTCGG-3’		
*bla*CTX-M-F	5’- ATG TGCAGYACCAGTAARGTKATGGC-3’	592	[15]
*bla*CTX-M-R	3’- TGGGTRAARTARGTSACCAGAAYSAGC GG-5’		

ESBL=Extended-spectrum beta-lactamase

### Statistical analysis

The data are presented descriptively in percentages displayed in tables.

## Results

In the present study, 51 isolates were isolated from broiler chickens and local chickens on EMBA. From these isolates, 39 samples were identified as *E. coli* based on the results of the PCR assay by detecting *usp*A gene sequences (Table-1) [12, 14, 15]. Sensitivity of *E. coli* to various antibiotics revealed disparate findings. All *E. coli* isolates were sensitive to IPM (100%), and 38 isolates were sensitive to FOX (97.4%). On average, *E. coli* isolates are sensitive to AMC (69.2%) and CRO (89.7%). These isolates were occasionally resistant to ENR (25.64%), followed by CN (20.51%), CRO (10.25%), AMC (7.69%), and FOX (2.56%). Table-2 presents the results of the sensitivity test. Four *E. coli* isolates derived from broiler chicken isolates that were tested using the antimicrobial susceptibility test were categorized as multidrug resistance (MDR) (Tables-3 and 4). MDR is defined as acquired resistance to at least one bacterial agent for three or more antibiotic groups [16].

**Table-2:** Results of antibiotic sensitivity test for 39 isolates of *Escherichia coli.*

Antibiotic	Disk content (μg/disk)	*Escherichia coli* isolates (n = 39)			Frequency of resistance (%)

		Sensitive	Intermediate	Resistant	
AMC	30	27	9	3	7.69
CRO	30	35	0	4	10.25
FOX	30	38	0	1	2.56
IPM	10	39	0	0	0
ENR	5	22	7	10	25.64
CN	10	31	0	8	20.51

AMC=Amoxicillin-clavulanic acid, CRO=Ceftriaxone, FOX=Cefoxitin, IPM=Imipenem, ENR=Enrofloxacin, CN=Gentamicin

**Table-3:** The results of antimicrobial resistance isolates based on chicken type.

Chickens	Multidrug resistance isolates based on chicken type (n = 39)			Frequency of resistance (%)

	n	Positive	Negative	
Local	20	0	20	0
Broiler	19	4	15	21.05

n=Total number of isolates

**Table-4:** The molecular detection results of ESBL gene.

Sample code	ESBL gene		

	*bla*TEM	*bla*CTX-M	*bla*SHV
Ab8	+	+	-
Ab9	+	+	-
Ab11	+	+	-
Ab12	+	+	-

ESBL=Extended-spectrum beta-lactamase

## Discussion

**Figure-1:**
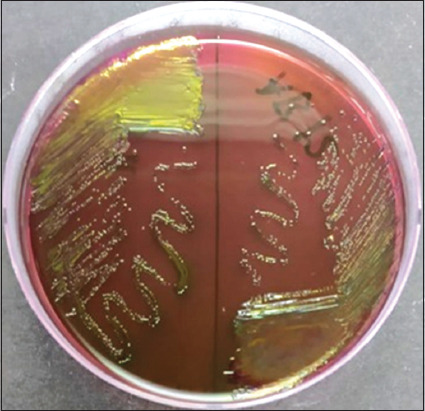
Cultured plates assumed as *Escherichia coli* colony on eosin methylene blue agar.

**Figure-2:**
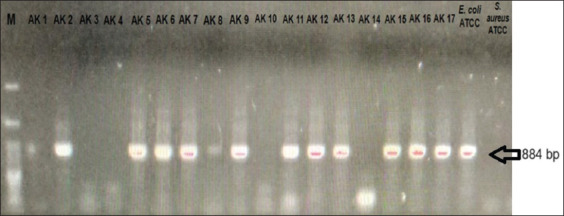
Gel electrophoresis visualization *usp*A gene of *Escherichia coli*.

Poultry is one of the most important sources of food in the world. Antimicrobial-resistant *E. coli* can be transmitted to humans from contaminated poultry products or by contact with poultry waste [17]. A total of 60 cloacal swabs from 30 broilers and 30 local chickens from Cibinong market, West Java, Indonesia were used as samples. Swabs were cultured on EMBA. The colony morphology of 51 isolates showed the growth of green metallic sheen with black centers, presumptively indicative of *E. coli* (Figure-1) [18]. All isolates were confirmed using PCR for molecular typing of the *usp*A target gene. Only 39 samples (76.5%) were identified as *E. coli*. These results are similar to those reported by Chen and Griffiths [19] and Godambe *et al*. [20], who used the *usp*A gene as a DNA marker for the identification of *E. coli* (Figure-2). In a previous study in Indonesia, the *usp*A gene was also used to identify *E. coli* from broilers [21]. The antimicrobial sensitivity test was performed on isolates identified as *E. coli* using the disk diffusion method. This study showed that *E. coli* had the highest resistance level against ENR, followed by CN and CRO, while AMC and FOX had much lower resistance levels. These results are consistent with several studies on antibiotic resistance in *E. coli* from chickens in Indonesia. Hardiati *et al*. [21] reported that *E. coli* from broilers are resistant to ENR and CN. The resistance of *E. coli* to ENR and CN was also similar to that reported in studies using broiler samples in Indonesia [22, 23]. Abo-Amer *et al*. [17] reported that *E. coli* isolated from chicken cloacae in Saudi Arabia were resistant to CN, AMC, and CRO; however, the resistance levels of CRO and AMC in this study were much lower than those reported by Lemlem *et al*. [24]. According to Ali *et al*. [25], *E. coli* isolated from chicken meats were resistant to CN but had a lower percentage of resistance to ENR and AMC. Aworh *et al*. [26] also reported resistance against CN and FOX in chickens. Resistance to AMC and CRO may be used to determine the activity of ESBL enzyme. In addition, there is an increased risk of infection with ESBL-producing bacteria associated with second- and third-generation cephalosporins [27, 28].

**Figure-3:**
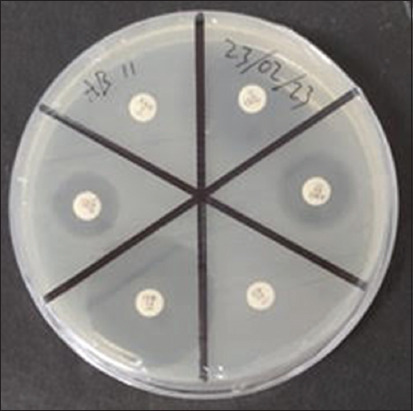
The antimicrobial susceptibility test result for *Escherichia coli* was performed on Mueller Hinton agar.

This study revealed that four isolates (10.2%) of *E. coli* from broilers were MDR (Figure-3), with three isolates resistant to four groups of antibiotics and one isolate resistant to three antibiotic groups. There have been many reports of AMR in broilers worldwide. This may be due to the overuse of antibiotics for disease prevention, to reduce bacterial invasion control, and as growth promoters to increase poultry production [22, 28]. The MDR observed in this study could be mediated by mobile genetic components, such as resistance genes [17]. Antimicrobials continue to be used outside the health-care system for various purposes and play an important role in animal production, particularly for veterinary and livestock purposes [29]. Furthermore, MDR is caused by uncontrolled antibiotic use, leading resistant bacteria to evolve through genetic mutations, swap genetic materials, and proliferation to fight against various antibiotics [30]. Antimicrobial-resistant *E. coli* may remain in the intestinal tracts of poultry for long periods, regardless of the use of antimicrobials, and can serve as a route for transmission to the human population by consumption [17].

Four MDR *E. coli* isolates were positive for the ESBL-encoding genes *bla*CTX-M and *bla*TEM, but negative for *bla*SHV. According to a study conducted by Lukman *et al*. [31], ESBL-producing *E. coli* was present in broiler feces in Bogor, Indonesia, which was related to the findings in this study. The results of this study are in accordance with several studies in Indonesia which reported that the ESBL-producing *E. coli* were identified from feces of broilers in Bogor reached 25% [32]; cloacal swabs of broilers from wet markets in Surabaya [23]; cloacal swab of layers in Blitar reached 7.03% [28]; swab cages and wastewater around broiler farm in Pasuruan 9.14% [33]; samples of chicken meat sold at Purwokerto’s market reached 7.14% [34]; from environmental samples of dairy farms in Yogyakarta, Probolinggo, and East Java [35–37]; and from companion dogs in Surabaya reached 9.41% [38]. These findings were also similar to those reported by Lemlem *et al*. [24], who detected *bla*CTX-M and *bla*TEM from broilers in Malaysia. Gundran *et al*. [39] also reported the detection of *bla*CTX-M and *bla*TEM in broilers from the Philippines. Numerous studies have reported that the same isolates have multiple beta-lactamase genes [38, 39]. Recently, *bla*CTX-M was identified as the leading ESBL gene in the world [24, 39]. The most commonly detected ESBL gene was *bla*CTX-M, followed by *bla*TEM and *bla*SHV [40]. The prevalence of ESBLs varies throughout the world, with Asian countries having the highest rates [41]. The results of this study suggest that local broiler farms that sell broilers at the Cibinong market are a source of ESBL-producing *E. coli*. The spread of ESBL-producing *E. coli* isolates from food animals without symptoms suggests that commensal *E. coli* may function as a reservoir for resistance genes and may constitute a danger of human transmission [39, 42].

ESBL-producing *E. coli* in this study showed that the prevalence of ESBL-producing *E. coli* in Indonesia is also increasing. A zoonotic disease may be caused by ESBL-producing bacteria in the environment, which may spread from animals to humans. Direct contact with the contaminated environment and consumption of contaminated meat are two ways to spread the disease. ESBL-producing *E. coli* can contaminate chicken meat if it is handled unhygienically during slaughter and handling if the chickens have the bacteria in their digestive tracts, or if it originates from a contaminated cage environment [33]. Several age groups, including pregnant women, children, the elderly, those with immunosuppression, as well as post-operative and chemotherapy patients, have a high potential for resistance to ESBL-producing *E. coli* [43]. ESBL-producing *E. coli* causes increased morbidity and mortality [15].

## Conclusion

The prevalence of MDR in *E. coli* isolates from broiler chickens and the discovery of ESBL genes (*bla*TEM and *bla*CTX-M) in the present study indicate that antibiotics are used more often in broiler chickens than in local chickens. Therefore, the use of antibiotics should be regulated and monitored regularly.

## Authors’ Contributions

SR: Planned and designed the study, sampling, analyzed the results, and drafted and revised the manuscript. IN: Conducted the study under the supervision of SR. IF: Analyzed the results and drafted and revised the manuscript. MM: Conducted the study. YDJ: Analyzed the results and drafted the manuscript. All authors have read, reviewed, and approved the final manuscript.

## References

[1] Rousham E.K, Unicomb L, Islam M.A (2018). Human, animal and environmental contributors to antibiotic resistance in low-resource settings:Integrating behavioural, epidemiological and one health approaches. Proc. Biol. Sci.

[2] Holmes A.H, Moore L.S.P, Sundsfjord A, Steinbakk M, Regmi S, Karkey A, Guerin P.J, Piddock L.J.V (2016). Understanding the mechanisms and drivers of antimicrobial resistance. Lancet.

[3] Nhung N.T, Cuong N.V, Thwaites G, Carrique-Mas J (2016). Antimicrobial usage and antimicrobial resistance in animal production in Southeast Asia:A review. Antibiotics (Basel).

[4] Murray C.J, Ikuta K.S, Sharara F, Swetschinski L, Aguilar G.R, Gray A, Tasak N (2022). Global burden of bacterial antimicrobial resistance in 2019:A systematic analysis. Lancet.

[5] Aarestrup F.M, Wegener H.C, Collignon P (2008). Resistance in bacteria of the food chain:Epidemiology and control strategies. Expert Rev. Anti Infect Ther.

[6] Untari T, Herawati O, Anggita M, Asmara W, Wahyuni A.E.T.H, Wibowo M.H (2021). The effect of antibiotic growth promoters (AGP) on antibiotic resistance and the digestive system of broiler chicken in Sleman, Yogyakarta. Bio Web. Conf.

[7] Founou L.L, Founou R.C, Essack S.Y (2016). Antibiotic resistance in the food chain:A developing country-perspective. Front. Microbiol.

[8] Noenchat P, Nhoonoi C, Srithong T, Lertpiriyasakulkit S, Sornplang P (2022). Prevalence and multidrug resistance of *Enterococcus* species isolated from chickens at slaughterhouses in Nakhon Ratchasima Province, Thailand. Vet. World.

[9] Siahaan S, Herman M.J, Fitri N (2022). Antimicrobial resistance situation in Indonesia:A challenge of multisector and global coordination. J. Trop Med.

[10] Parathon H, Kuntaman K, Widiastoety T.H, Muliawan B.T, Karuniawati A, Qibtiyah M, Djanun Z, Tawilah J.F, Aditama T, Thamlikitkul V, Vong S (2017). Progress towards antimicrobial resistance containment and control in Indonesia. BMJ.

[11] World Health Organization (2021). Global Antimicrobial Resistance and Use Surveillance System (GLASS) Report.

[12] Indrawati A, Khoirani K, Setiyaningsih S, Affif U, Ningrum S.G (2021). Detection of tetracycline resistance genes among *Escherichia coli* isolated from layer and broiler breeders in West Java, Indonesia. Trop. Anim. Sci. J.

[13] Lewis J.S, Weinstein M.P, Bobenchik A.M (2022). Performance Standards for Antimicrobial Susceptibility Testing. 32^nd^ ed., Vol. M100-Ed32. Clinical and Laboratory Standards Institute, Wayne, PA.

[14] Weill F.X, Demartin M, Tandé D, Espié E, Rakotoarivony I, Grimont P.A (2004). SHV-12-like extended-spectrum-b-lactamase-producing strains of *Salmonella enterica* serotypes Babelsberg and Enteritidis isolated in France among infants adopted from Mali. J. Clin. Microbiol.

[15] Rottier W.C, Ammerlaan H.S, Bonten M.J (2012). Effects of confounders and intermediates on the association of bacteraemia caused by extended-spectrum b-lactamase-producing *Enterobacteriaceae* and patient outcome:A meta-analysis. J. Antimicrob. Chemother.

[16] Basak S, Singh P, Rajurkar M (2016). Multidrug-resistant and extensively drug-resistant bacteria:A study. J. Pathog.

[17] Abo-Amer A.E, Shobrak M.Y, Altalhi A.D (2018). Isolation and antimicrobial resistance of *Escherichia coli* isolated from farm chickens in Taif, Saudi Arabia. J. Glob. Antimicrob. Resist.

[18] Leboffe M.J, Pierce B.E (2021). A Photographic Atlas for the Microbiology Laboratory.

[19] Chen J, Griffiths M.W (1998). PCR differentiation of *Escherichia coli* from other Gram-negative bacteria using primers derived from the nucleotide sequences flanking the gene encoding the universal stress protein. Lett. Appl. Microbiol.

[20] Godambe L.P, Bandekar J, Shashidhar R (2017). Species-specific PCR based detection of *Escherichia coli* from Indian foods. 3 Biotech.

[21] Hardiati A, Safika S, Wibawan I.W.T, Indrawati A, Pasaribu F.H (2021). Isolation and detection of antibiotics resistance genes of *Escherichia coli* from broiler farms in Sukabumi, Indonesia. J. Adv. Vet. Anim. Res.

[22] Purwanto E, Marmansari D, Sari D.K, Hatta M (2019). Antibiotic resistance of *E. coli* isolates from broiler Chick's cecum in Makassar City. J. Ris. Vet. Indones.

[23] Effendi M.H, Tyasningsih W, Yurianti Y.A, Rahmahani J, Harijani N, Plumeriastuti H (2021). Presence of multidrug resistance (MDR) and extended-spectrum beta-lactamase (ESBL) of *Escherichia coli* isolated from cloacal swab of broilers in several wet markets in Surabaya, Indonesia. Biodiversitas.

[24] Lemlem M, Aklilu E, Mohammed M, Kamaruzzaman F, Zakaria Z, Harun A, Devan S.S (2023). Molecular detection and antimicrobial resistance profiles of Extended-Spectrum Beta-Lactamase (ESBL) producing *Escherichia coli* in broiler chicken farms in Malaysia. PLoS One.

[25] Ali S.S, Sonbol F.I, Sun J, Hussein M.A, Hafez A.E.E, Abdelkarim E.A, Kornaros M, Ali A, Azab M (2020). Molecular characterization of virulence and drug resistance genes-producing *Escherichia coli* isolated from chicken meat:Metal oxide nanoparticles as novel antibacterial agents. Microb. Pathog.

[26] Aworh M.K, Kwaga J, Okolocha E, Harden L, Hull D, Hendriksen R.S, Thakur S (2020). Extended-spectrum ß-lactamase-producing *Escherichia coli* among humans, chickens and poultry environments in Abuja, Nigeria. One Health Outlook.

[27] Amin M, Wasito E.B, Triyono E.A (2020). Comparison between exposure of ciprofloxacin and cefotaxime on developing of *Escherichia coli* ESBL. Folia Med. Indones.

[28] Wibisono F.J, Sumiarto B, Untari T, Effendi M.H, Permatasari D.A, Witaningrum A.M (2020). The presence of extended-spectrum beta-lactamase (ESBL) producing *Escherichia coli* on layer chicken farms in Blitar Area, Indonesia. Biodiversitas.

[29] Kimera Z.I, Mshana S.E, Rweyemamu M.M, Mboera L.E, Matee M.I (2020). Antimicrobial use and resistance in food-producing animals and the environment:An African perspective. Antimicrob. Resist. Infect. Control.

[30] Velazquez-Meza M.E, Galarde-López M, Carrillo-Quiróz B, Alpuche-Aranda C.M (2022). Antimicrobial resistance:One health approach. Vet. World.

[31] Lukman D.W, Sudarwanto M.B, Purnawarman T, Latif H, Pisestyani H, Sukmawinata E, Akineden Ö (2016). CTX-M-1 and CTX-M-55 producing *Escherichia coli* isolated from broiler feces in poultry slaughterhouse, Bogor, West Java Province. Glob. Adv. Res. J. Med. Med. Sci.

[32] Masruroh C.A, Sudarwanto M.B, Latif H (2016). The occurrence of extended-spectrum Β-Lactamase-producing *Escherichia col*i from broiler feces in Bogor [Tingkat kejadian *Escherichia col*i penghasil extended spectrum β-Lactamase yang diisolasi dari feses broiler di kota Bogor]. J. Sain Vet.

[33] Yanestria S.M, Dameanti F.N.A.E.P, Musayannah B.G, Pratama J.W.A, Witaningrum A.M, Effendi M.H, Ugbo E.N (2022). Antibiotic resistance pattern of Extended-Spectrum b-Lactamase (ESBL) producing *Escherichia coli* isolated from broiler farm environment in Pasuruan district, Indonesia. Biodiversitas.

[34] Widhi A.P.K.N, Saputra I.N.Y (2021). Antibiotic residues and the presence of ESBL-producing *Escherichia col*i on broiler chicken meat at the Purwokerto city market. J. Kes. Ling. Indones.

[35] Dameanti F.N.A.E.P, Yanestria S.M, Widodo A, Effendi M.H, Plumeriastuti H, Tyasningsih W, Sutrisno R, Akramsyah M. A (2023). Incidence of *Escherichia coli* producing Extended-spectrum beta-lactamase in wastewater of dairy farms in East Java, Indonesia. Biodiversitas.

[36] Maulana K.Y, Pichpol D, Farhani N.R, Widiasih D.A, Unger F, Punyapornwithaya V, Meeyam T (2021). Antimicrobial resistance characteristics of Extended Spectrum Beta Lactamase (ESBL)-producing *Escherichia coli* from dairy farms in the Sleman district of Yogyakarta province, Indonesia. Vet. Integr. Sci.

[37] Widodo A, Lamid M, Effendi M.H, Khailrullah A.R, Riwu K.H.P, Yustinasari L.R, Kurniawan S.C, Ansori A.N.M, Silaen O.S.M, Dameanti F.N.A.E.P (2022). Antibiotic sensitivity profile of multidrug-resistant (MDR) *Escherichia coli* isolated from dairy cow's milk in Probolinggo, Indonesia. Biodiversitas.

[38] Kristianingtyas L, Effendi M.H, Witaningrum A.M, Wardhana D.K, Ugbo E.N (2021). Prevalence of extended-spectrum ß-lactamase-producing *Escherichia coli* in companion dogs in animal clinics, Surabaya, Indonesia. Intl. J. One Health.

[39] Gundran R.S, Cardenio P.A, Villanueva M.A, Sison F.B, Benigno C.C, Kreausukon K, Pichpol D, Punyapornwithaya V (2019). Prevalence and distribution of *bla*_CTX-M_, *bla*_SHV_, bla*_TEM_* genes in extended-spectrum b-lactamase-producing *E. coli* isolates from broiler farms in the Philippines. BMC Vet. Res.

[40] Jena J, Sahoo R.K, Debata N.K, Subudhi E (2017). Prevalence of TEM, SHV, and CTX-M genes of extended-spectrum b-lactamase-producing *Escherichia coli* strains isolated from urinary tract infections in adults. 3 Biotech.

[41] Hawkey P.M (2008). Prevalence and clonality of extended-spectrum b-lactamases in Asia. Clin. Microbiol. Infect.

[42] Li S, Zhao M, Liu J, Zhou Y, Miao Z (2016). Prevalence and antibiotic resistance profiles of extended-spectrum b-lactamase–producing *Escherichia coli* isolated from healthy broilers in Shandong Province, China. J. Food Prot.

[43] Franz E, Veenman C, van Hoek A.H, Husman A.D.R, Blaak H (2015). Pathogenic *Escherichia coli* producing Extended-Spectrum b-Lactamases isolated from surface water and wastewater. Sci. Rep.

